# Comparison of the Efficacy of Deep Brain Stimulation in Different Targets in Improving Gait in Parkinson's Disease: A Systematic Review and Bayesian Network Meta-Analysis

**DOI:** 10.3389/fnhum.2021.749722

**Published:** 2021-10-22

**Authors:** Tianyi Chen, Fabin Lin, Guoen Cai

**Affiliations:** ^1^School of Mathematics, Shandong University, Jinan, China; ^2^Department of Neurology, Fujian Medical University Union Hospital, Fuzhou, China; ^3^Fujian Key Laboratory of Molecular Neurology, Institute of Clinical Neurology, Institute of Neuroscience, Fujian Medical University, Fuzhou, China

**Keywords:** Parkinson's disease, deep brain stimulation, Bayesian network meta-analysis, gait, motor symptoms

## Abstract

**Background:** Although a variety of targets for deep brain stimulation (DBS) have been found to be effective in Parkinson's disease (PD), it remains unclear which target for DBS leads to the best improvement in gait disorders in patients with PD. The purpose of this network meta-analysis (NMA) is to compare the efficacy of subthalamic nucleus (STN)-DBS, internal globus pallidus (GPi)-DBS, and pedunculopontine nucleus (PPN)-DBS, in improving gait disorders in patients with PD.

**Methods:** We searched the PubMed database for articles published from January 1990 to December 2020. We used various languages to search for relevant documents to reduce language bias. A Bayesian NMA and systematic review of randomized and non-randomized controlled trials were conducted to explore the effects of different targets for DBS on gait damage.

**Result:** In the 34 included studies, 538 patients with PD met the inclusion criteria. The NMA results of the effect of the DBS “on and off” on the mean change of the gait of the patients in medication-off show that GPi-DBS, STN-DBS, and PPN-DBS are significantly better than the baseline [GPi-DBS: –0.79(–1.2, –0.41), STN-DBS: –0.97(–1.1, –0.81), and PPN-DBS: –0.56(–1.1, –0.021)]. According to the surface under the cumulative ranking (SUCRA) score, the STN-DBS (SUCRA = 74.15%) ranked first, followed by the GPi-DBS (SUCRA = 48.30%), and the PPN-DBS (SUCRA = 27.20%) ranked last. The NMA results of the effect of the DBS “on and off” on the mean change of the gait of the patients in medication-on show that, compared with baseline, GPi-DBS and STN-DBS proved to be significantly effective [GPi-DBS: –0.53 (–1.0, –0.088) and STN-DBS: –0.47(–0.66, –0.29)]. The GPi-DBS ranked first (SUCRA = 59.00%), followed by STN-DBS(SUCRA = 51.70%), and PPN-DBS(SUCRA = 35.93%) ranked last.

**Conclusion:** The meta-analysis results show that both the STN-DBS and GPi-DBS can affect certain aspects of PD gait disorder.

## 1. Introduction

Parkinson's disease (PD) is currently the second most prevalent neurodegenerative disease worldwide. Gait disorders widely and severely affect patients with PD as they significantly limit the ability of the patient to walk and often cause falls and fall-related injuries. In addition, with the progression of the disease, their frequency and severity gradually increase (Nonnekes et al., 2015, 2019). Dopamine therapy and surgery are commonly used to treat gait disorders in patients with early and mid-stage PD, but their beneficial effect is minimal for patients with advanced PD (Gazewood et al., 2013). Deep brain stimulation (DBS) is a novel treatment for advanced PD-related gait disorders (Ferraye et al., 2008). Compared with the traditional treatments for PD, DBS has the advantages of reversibility, preservation of neuronal tissue, and adjustability of the treatment plan according to the disease state of the patient. It is believed that the subthalamic nucleus (STN) (Remple et al., 2011; Jahanshahi et al., 2015), internal globus pallidus (GPi) (Okun, 2012), and pedunculopontine nucleus (PPN) are the stimulation targets for improving gait disorders in patients with advanced PD.

Some randomized controlled trials were unable to implement two specific interventions, resulting in the inability to obtain direct evidence from face-to-face trials. As a result, it can be challenging to evaluate the effectiveness of many clinically indicated interventions available and determine the best intervention (Nikolakopoulou et al., 2018). Through network-meta-analysis (NMA), inferences can be made about every possible comparison between a pair of interventions in the network, even if some comparisons have never been evaluated in actual trials (Bafeta et al., 2013; Dias and Caldwell, 2019). Ultimately, we can combine the direct and indirect comparisons and determine the best intervention.

The treatment of gait disorders is a challenge. Realizing that few studies directly compare the efficacy of DBS with different stimulation targets, we conducted an NMA to evaluate the potential effects of STN-DBS, GPi-DBS, and PPN-DBS in the treatment of PD gait.

The Unified Parkinson's Disease Rating Scale (UPDRS) is currently the most widely used clinical grading system for PD (Movement Disorder Society Task Force on Rating Scales for Parkinson's Disease, 2003). This study evaluates the 3.29-step item of gait in the UPDRS. Therefore, our goal is to compare and rank the therapeutic effects of these three types of interventions on PD gait.

## 2. Method

This NMA is implemented in accordance with the Preferred Reporting Items for Systematic Reviews and Meta-Analyses extension statement for NMA.

### 2.1. Search Method

We searched the PubMed database for articles published from January 1990 to December 2020 and searched for relevant documents in various languages to reduce language bias. However, ultimately, only documents in English were considered appropriate. After that, we also screened the references of the retrieved articles to determine related research studies.

### 2.2. Eligibility Criteria

1. Clinical trials of using DBS to treat idiopathic PD.
2. Research object: patients clinically diagnosed with PD.
3. Results: studies used the UPDRS III and UPDRS III item 29 to evaluate therapeutic efficacy.
4. Including one or more of the following three surgical methods: STN-DBS, GPi-DBS, and PPN-DBS.

### 2.3. Exclusion Criteria

1. Clinical trials of DBS in the treatment of diseases other than PD.
2. No clinically controlled trials were conducted at the same time.
3. Lost data or data that cannot be extracted.
4. Studies used the MDS-UPDRS III and MDS-UPDRS III item 29 to evaluate therapeutic efficacy.

### 2.4. Quality Assessment and Data Extraction

The Cochrane Collaboration's risk-of-bias tool was used to assess the quality of previous systematic reviews. The quality of each manuscript was evaluated by two researchers and then discussed with the main researcher to reach an agreement. We extracted the following variables from the collected manuscripts: name of the first author, date of publication, number of participants, age of participants, intervention measures, sex ratio, disease duration, post-surgery duration, and funding.

### 2.5. Effective Measurement

The UPDRS is currently the most commonly used clinical grading scale system for PD. The UPDRS III score is the main evaluation index for PD therapeutic research, and the UPDRS III gait sub-score is defined as the UPDRS III item 29 score. Therefore, we used the UPDRS III item 29 to evaluate the improvement of gait in patients with PD. In addition, the UPDRS III total score was used to assess the improvement of motor symptoms in patients with PD.

### 2.6. Statistical Analysis

In this study, the R language (R program software V.3.5.3, CRAN Project) was used to conduct NMA in order to compare the efficacy of different therapies. The specific method refers to our previous study (Lin et al., 2021). In short, NMA was performed based on the Bayesian framework, using the Markov Chain Monte Carlo method in the R software, including 4 chains with over-dispersed initial values and Gibbs sampling based on 50,000 iterations after 20,000 aging stages. The mean difference and 95% CI of the difference were obtained, and the significance level was 0.05. Additionally, the rank probability of each clinical outcome was assessed. The deviation information criterion (DIC) was used to evaluate the goodness of fit in this study. The quality of the model is negatively correlated with the value of DIC. By comparing the DIC values between the models, the suitability of the models can be assessed (Carpinella et al., 2007). To be able to determine whether small research effects exist, we examined each result and compared the adjusted funnel chart. In this study, we drew a funnel chart of the mean difference between all comparisons after treatment and baseline.

## 3. Results

### 3.1. Research Description

**Figure 1:**
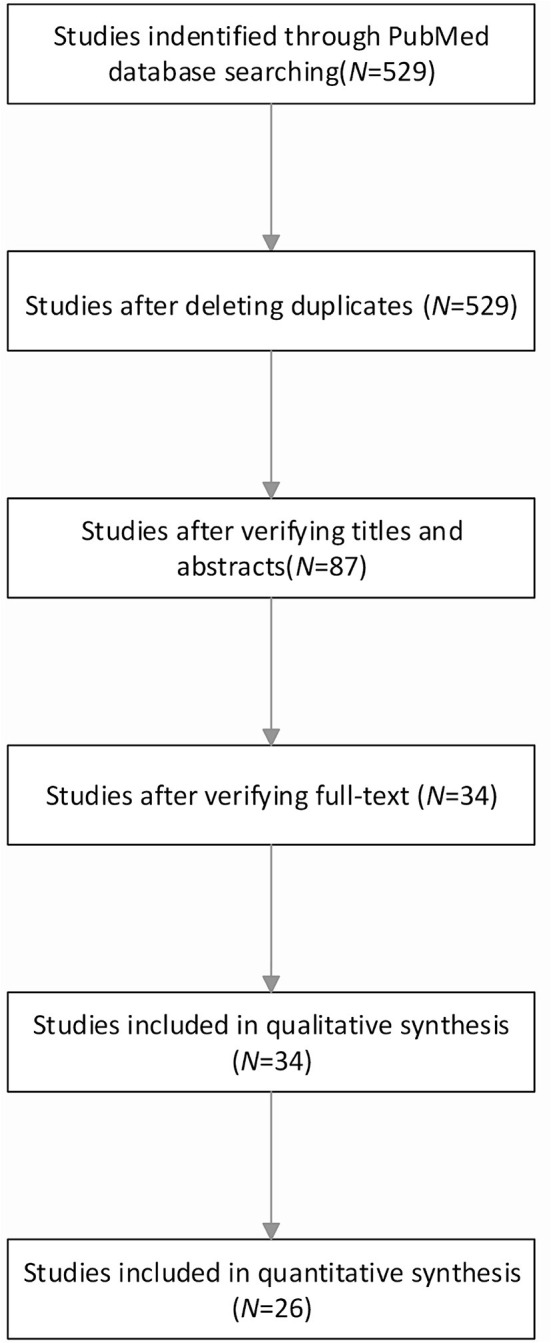
Flowchart of study selection.

**Figure 2:**
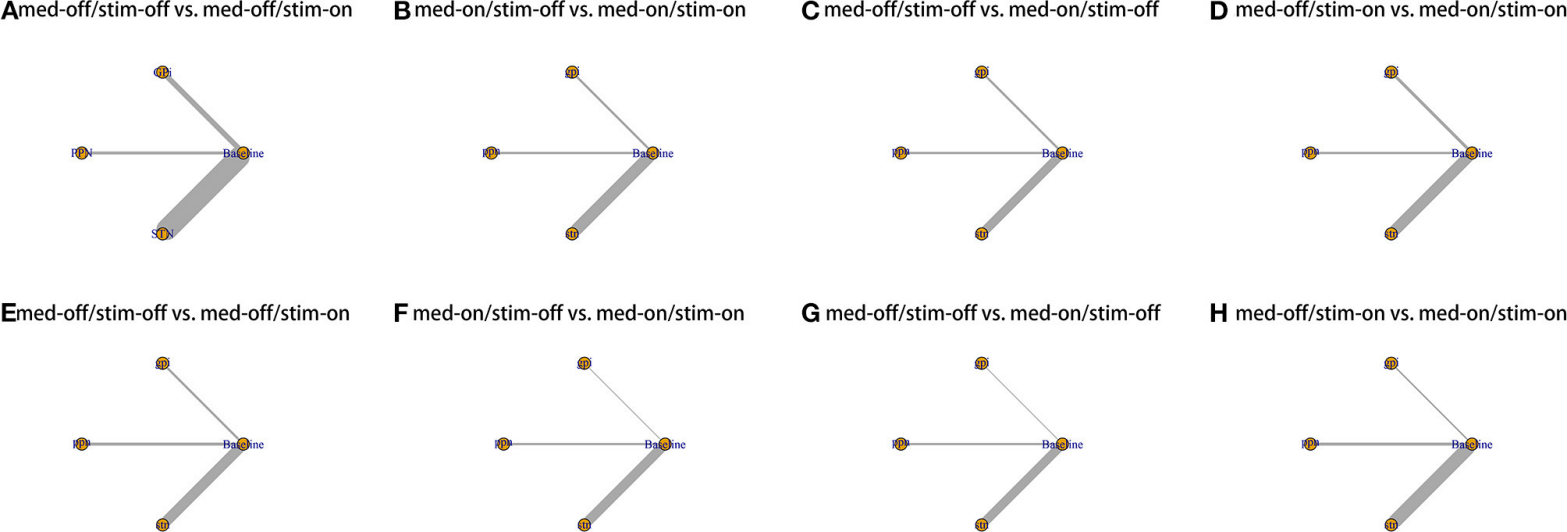
Network plot for all studies. **(A)** UPDRS III-Gait med-off/stim-off vs. med-off/stim-on. **(B)** UPDRS III-Gait med-on/stim-off vs. med-on/stim-on. **(C)** UPDRS III-Gait med-off/stim-off vs. med-on/stim-off. **(D)** UPDRS III-Gait med-off/stim-on vs. med-on/stim-on. **(E)** UPDRS III- Total med-off/stim-off vs. med-off/stim-on. **(F)** UPDRS III- Total med-on/stim-off vs. med-on/stim-on. **(G)** UPDRS III- Total med-off/stim-off vs. med-on/stim-off. **(H)** UPDRS III- Total med-off/stim-on vs. med-on/stim-on.

**Figure 3:**
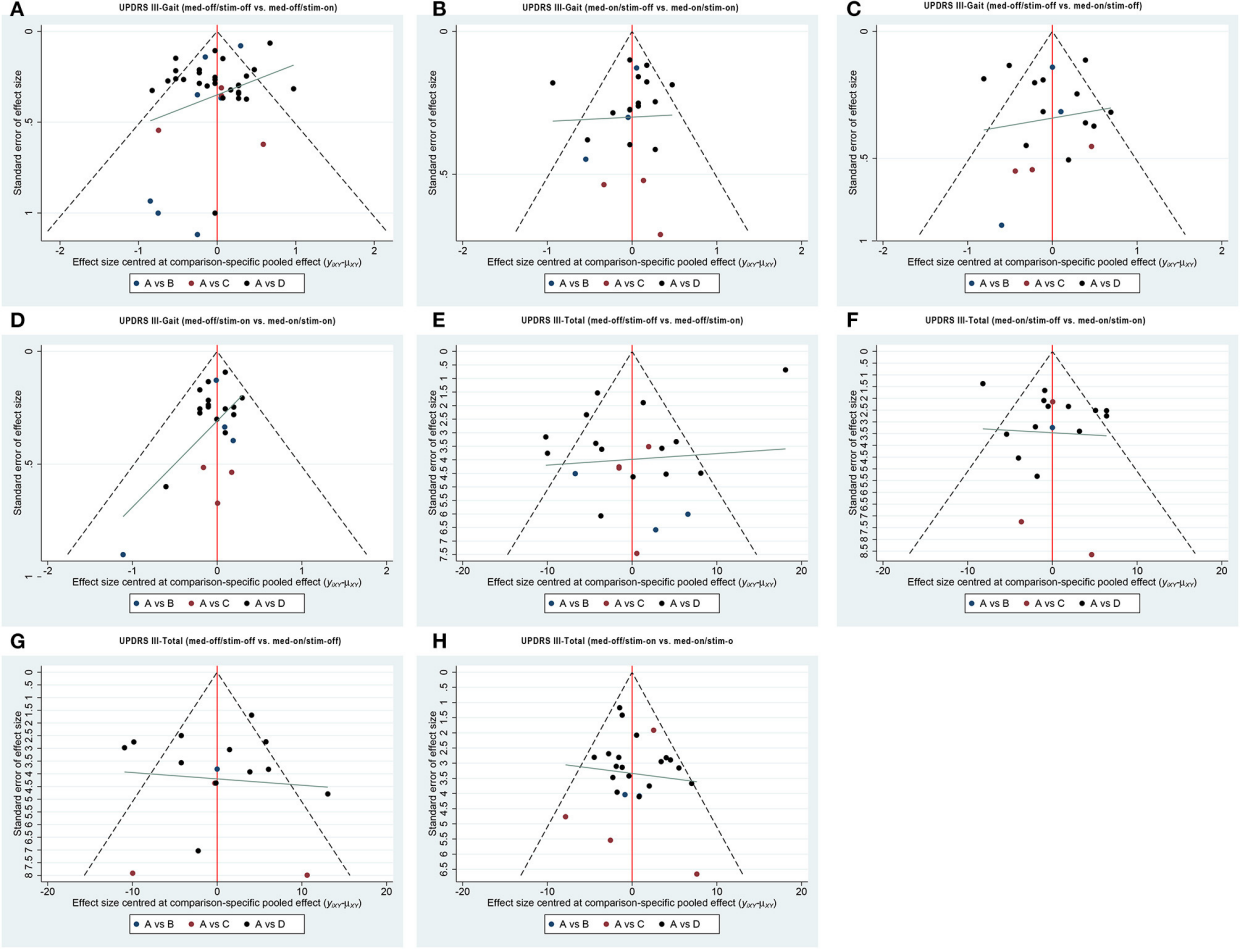
Comparison-adjusted funnel plot for the network of functional outcome; **(A)** UPDRS III-Gait (med-off/stim-off vs. med-off/stim-on), **(B)** UPDRS III-Gait (med-on/stim-off vs.med-on/stim-on), **(C)** UPDRS III-Gait (med-off/stim-off vs. med-on/stim-off), **(D)** UPDRS III-Gait (med-off/stim-on vs. med-on/stim-on). **(E)** UPDRS III-Total (med-off/stim-off vs. med-off/stim-on), **(F)** UPDRS III- Total (med-on/stim-off vs.med-on/stim-on), **(G)** UPDRS III- Total (med-off/stim-off vs. med-on/stim-off), **(H)** UPDRS III- Total (med-off/stim-on vs. med-on/stim-on).

**Figure 4:**
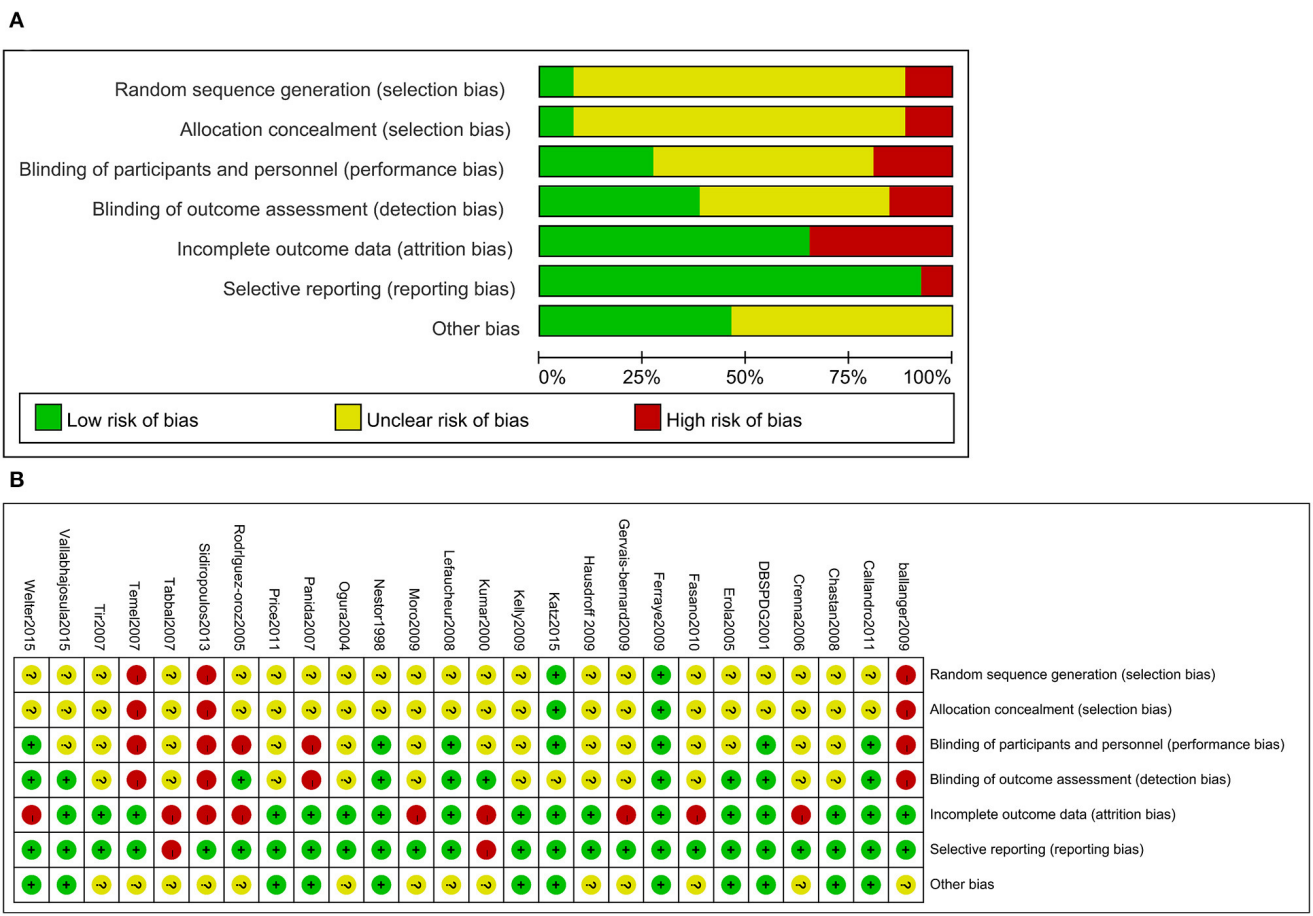
Risk of bias for the included trials **(A)**. Risk of bias summary for the included trials **(B)**.

The basic process of research data collection is illustrated in Figure 1. The network plot of the overall efficacy is shown in Figure 2. The basic information of the data of the study is shown in Table 1. The comparison-adjusted funnel plot for the network of the functional outcome is shown in Figure 3 and the risk of bias for the included trials is shown in Figure 4. In our first search, we identified 529 articles and subsequently eliminated 442 irrelevant articles based on the title and abstract of the article. Then, we carefully reviewed the full text of the remaining 87 articles and eliminated 53 of them. Our exclusion criteria were as follows: no extracted data; no available data; inappropriate diagnosis, and comment/overview. Ultimately, this study included a total of 34 studies and 26 of which were included in quantitative studies (Gálvez-Jiménez et al., 1998; Kumar et al., 1999; Obeso et al., 2001; Ogura et al., 2004; Erola et al., 2005; Rodriguez-Oroz et al., 2005; Crenna et al., 2006; Piboolnurak et al., 2007; Tabbal et al., 2007; Temel et al., 2007; Tir et al., 2007; Chastan et al., 2008; Lefaucheur et al., 2008; Ballanger et al., 2009; Ferraye et al., 2009; Gervais-Bernard et al., 2009; Moro et al., 2009; Schneider et al., 2009; Fasano et al., 2010; Kelly et al., 2010; Caliandro et al., 2011; Price et al., 2011; Sidiropoulos et al., 2013; Katz et al., 2015; Vallabhajosula et al., 2015; Welter et al., 2015).

**Table 1:** Characteristics of included studies.

**Number**	**Intervention**	**Author&Year**	**Sample** **size**	**Age** **(mean (SD) y)**	**Gender** **(M/F)**	**Disease** **duration** **(y ears)**	**Post** **surgery** **duration**	**Funding**
1	STN-DBS	Tir, 2007	100	58.7 ± 8.2	55/45	13.6 ± 4.4	12 m	/
2	single electrode guided STN-DBS or multiple electrode guided STN-DBS	Temel, 2007	55 (S:32 M:23)	61.6 (S:59.4 ± 7 M:64.6 ± 9.6)	36/19 (S:21/11 M:15/8)	12.3 (S:13.1 ± 5.1 M:11.3 ± 5.6)	12 m	/
3	STN-DBS	Tabbal, 2007	72	63 ± 8.2	41/31	14.5 ± 6.5	6 m	The Sam and Barbara Murphy Fund, the Elliot H. Stein Family Fund
4	STN-DBS	Simuni, 2002	12	58 ± 11	10/2	12 ± 4	12 m	/
5	STN-DBS	Rodrlguez-oroz, 2005	69 (S:49 M:20)	58.6 (S:59.8 ± 9.8 G:55.8 ± 9.4)	38/31 (S:25/24 G:13/7)	14.2 (S:14.1 ± 5.9 G:14.4 ± 5.7)	3–4 y	Medtronic Europe
6	STN-DBS	Panida, 2007	33	53.4 ± 8.3	24/9	/	5y	/
7	GPi-DBS	Ogura, 2004	30	57.7	16/14	8.4	12 m	/
8	GPi-DBS (There is an example of GPi+VIM)	Nestor, 1998	5	63.2 ± 7.5	4/1	10.2 ± 4.7	3 m	Medtronic, Minneapolis, MN, the National Parkinson Foundation, Miami, Fl and the Parkinson Foundation of Canada
9	STN-DBS	Lefaucheur, 2008	54	59	34/20	14	12 m	/
10	STN-DBS	Erola, 2005	29	60 ± 8	20/9	13 ± 7	12m	Finnish Parkinson Foundation
11	STN-DBS or GPi-DBS	DBSPDG, 2001	134 (S:96 G:38)	58.1 (S:59.0 ± 9.6 G:55.7 ± 9.8)	87/37 (S:60/36 G:27/11)	/	6m	/
12	STN-DBS	Crenna, 2006	10	60.2 ± 4.8	5/5	16.9 ± 5.5	10.4 ± 7 m	Italian Ministry of Health
13	STN-DBS or GPi-DBS	Burchiel, 1999	10 (S:6 G:4)	56 6 13 (S:62.8 ± 12 G:46.5 ± 11)	7/3	12.4 (S:13.6 ± 5 G:10.6 ± 2)	3m	United States Public Health Service
14	PPNa-DBS	Welter, 2015	4	62 ± 9.5	1/3	15.8 ± 5.1	6m	The Institut National de la Recherche Me'dicale (INSERM), the 'Institut du Cerveau et de la Moelle Epinie're' (ICM) Foundation, the 'Re'gie Autonome des Transports Parisiens' (RATP), the 'Fondation pour la Recherche Medicale' (FRM) and the programme 'Investissements d'avenir' (ANR-10-IAIHU-06)
15	STN-DBS	Vallabhajosula, 2015	19	61.8 ± 9	16/3	13.6 ± 4.2	/	The National Parkinson Foundation Center of Excellence, the UF Foundation, and UF Center for Movement Disorders and Neurorestoration
16	STN-DBS	Stegemoller, 2013	17	61.5 ± 9.2	14/3	13.6 ± 3.9	30.5 ± 19.2 m	The National Parkinson Foundation UF Center for Excellence and y NIH grant 5R03HD054594-02
17	STN-DBS	Sidiropoulos, 2013	45	59.5 ± 7.8	35/10	17.8 ± 5.7	4y	/
18	STN-DBS	Romito, 2009	20	56.4 ± 6.9	11/9	14.3 ± 6.2	5y	The Italian Ministry of University and Research (National Interest Project number 2001062543 to AA)
19	STN-DBS or GPi-DBS	Price, 2011	37	58.8 ± 7	28/9	12.4	4m	NINDS K23NS060660 (CP), NIH T35 07489 (CF), UF National Parkinson Foundation Center of Excellence and UF Foundation
20	STN-DBS	Phibbs, 2013	20	62	16/4	12.5	3y	NIH grant 1UL 1RR024975 NCRR and grant UL1 TR000445 from NCATS/NIH
21	STN-DBS	Nardo, 2014	9	66.4 ± 6.0	7/2	3.1 ± 1.3	3.3 ± 1.2 y	/
22	PPN-DBS	Moro, 2009	6	65.2 ± 2	5/1	15.5 ± 6.2	12m	The National Parkinson Alliance
23	STN-DBS and PPN-DBS	Moreau, 2009	4	/	/	/	/	/
24	STN-DBS	Kelly, 2009	8	51.9 ± 8.7	6/2	10.1 ± 3.5	/	The National Institutes of Health grant HD-007424 and a grant from Medtronic
25	STN-DBS or GPi-DBS	Katz, 2015	235 (S:108 G:127)	60.9	199/36	11.8	2 y	The Cooperative Studies Program of the Department of Veterans Affairs Office of Research and Development, the National Institute of Neurological Disorders and Stroke, and Medtronic
26	STN-DBS	Hausdorff, 2009	13	63.6 ± 8.7	10/3	12.9 ± 5.6	12 ± 7 m	NIH (AG-14100), the Israel Ministry of Absorption, the European Union Sixth Framework Program (FET018474-2, Dynamic Analysis of Physiological Networks, DAPHNet, STREP 045622 SENSing, ect)
27	STN-DBS	Gervais-bernard, 2009	23	55.1 ± 7.2	17/6	12.9 ± 3.2	5y	/
28	STN-DBS and PPNa-DBS	Ferraye, 2009	6	63.3 ± 6.8	4/2	20.7 ± 7.1	1y	The Michael J. Fox Foundation, the Fondation de France, the Centre Hospitalier Universitaire de Grenoble, project FREESTIPP and Medtronic
29	STN-DBS	Fasano, 2010	20	56.9 ± 7.2	12/8	13.7 ± 4.8	8y	NEURECA onlus
30	STN-DBS and SNr-DBS	Chastan, 2008	7	61 ± 7	5/2	18.3 ± 4.2	43.6 ± 20.1 m	INSERM
31	STN-DBS	Chan 2013,	46	/	36/10	/	/	St. Jude Medical, CurePSP, CIHR and Medtronic
32	PPT-DBS	Callandro, 2011	3	66	3/0	12.3 ± 1.7	/	/
33	STN-DBS	Burrows, 2012	11	64 ± 7	8/3	16 ± 5	/	American Parkinson's Disease Association and Medtronic
34	PPN-DBS	Ballanger	3	67.3 ± 2.1	3/0	21.7 ± 3.4	15.3 ± 8.1	CIHR New Investigator Research Award and CIHR-Industry Sponsored Investigator Award

### 3.2. Unified Parkinson's Disease Rating Scale III-Gait (Medication-Off/Stimulation-Off vs. Medication-Off/Stimulation-On

**Figure 5:**
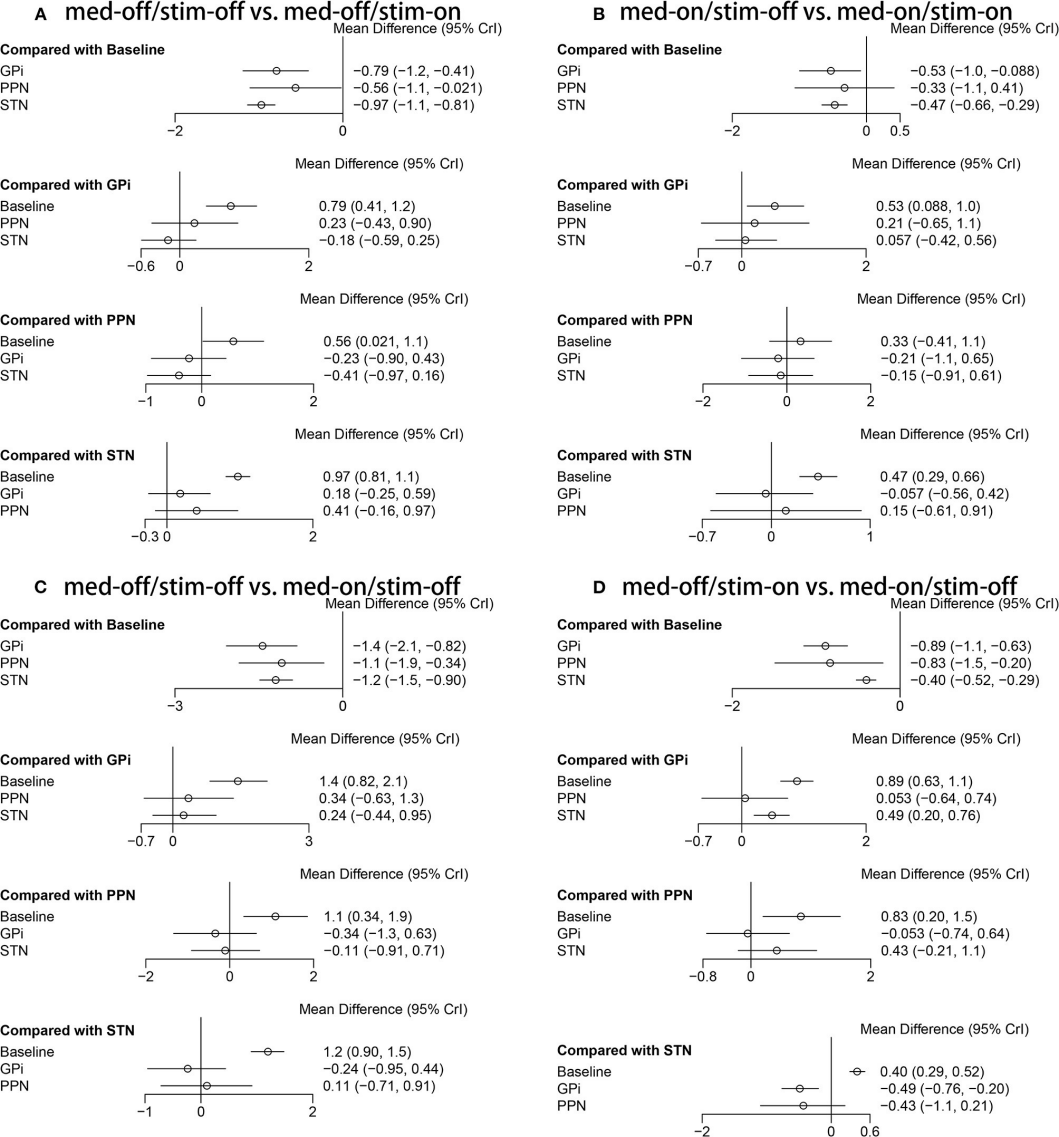
Forest plot of mean difference of UPDRS-III (29 item, gait); **(A)** med-off/stim-off vs. med-off/stim-on, **(B)** med-on/stim-on vs. med-on/stim-on, **(C)** med-off/stim-off vs. med-on/stim-off, **(D)** med-off/stim-on vs. med-on/stim-on.

**Figure 6:**
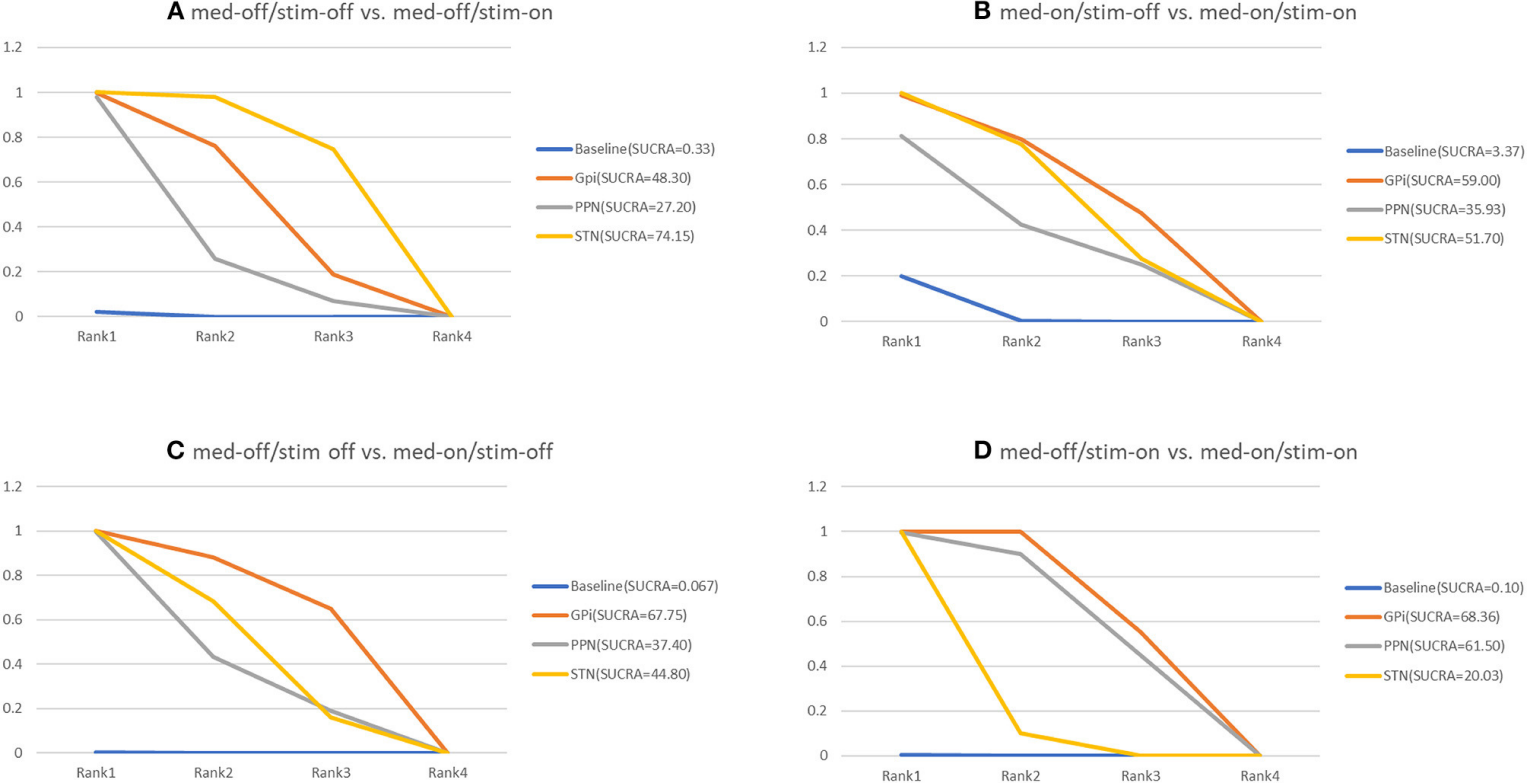
Cumulative rank of three surgical intervention of UPDRS-III (29 item, gait); **(A)** med-off/stim-off vs. med-off/stim-on, **(B)** med-on/stim-on vs. med-on/stim-on, **(C)** med-off/stim-off vs. med-on/stim-off, **(D)** med-off/stim-on vs. med-on/stim-on.

The NMA results of the effect of the stimulation “on and off” on the mean change of the gait of the patients in medication-off are summarized in Figure 5. In addition, Figure 6 shows the surface under the cumulative ranking (SUCRA). The comparison results of the NMA show that GPi-DBS, STN-DBS, and PPN-DBS are superior to the baseline [GPi-DBS: –0.79(–1.2, –0.41), STN-DBS: –0.97(–1.1, –0.81), and PPN-DBS: –0.56(–1.1, –0.021)]. According to the results of the SUCRA scores, STN-DBS (SUCRA = 74.15%) ranks first, followed by GPi-DBS (SUCRA = 48.30%), and PPN-DBS (SUCRA = 27.20%) ranks last.

### 3.3. Unified Parkinson's Disease Rating Scale III-Gait (Medication-On/Stimulation-Off vs. Medication-On/Stimulation-On)

The NMA results of the effect of the stimulation “on and off” on the mean change of the gait of the patients in medication-on are shown in Figure 5 and the SUCRA is shown in Figure 6. Compared with the baseline, the GPi-DBS and STN-DBS proved to be significantly effective [GPi-DBS: –0.53(–1.0, –0.088) and STN-DBS: –0.47(–0.66, –0.29)]. The SUCRA scores reveal the rank of the three surgical interventions as follows: The GPi-DBS (SUCRA = 59.00%) ranks first, followed by STN-DBS (SUCRA = 51.70%), and PPN-DBS (SUCRA = 35.93%) ranks last.

### 3.4. Unified Parkinson's Disease Rating Scale III-Gait (Medication-Off/Stimulation-Off vs. Medication-On/Stimulation-Off)

The NMA results show the effect of medication “on and off” on the mean change of the gait of the patients in stimulation-off (Figure 5), the SUCRA is shown in Figure 6. The GPi-DBS, STN-DBS, and PPN-DBS show effective improvement compared to the baseline [GPi-DBS: –1.4(–2.1, –0.82), STN-DBS: –1.2(–1.5, –0.90), and PPN-DBS: –1.1(–1.9, –0.34)]. The rank of the three surgical interventions is that based on the SUCRA scores that is as follows: The GPi-DBS (SUCRA = 67.75%) ranks first, followed by STN-DBS (SUCRA = 44.80%), and PPN-DBS (SUCRA = 37.40%) ranks last.

### 3.5. Unified Parkinson's Disease Rating Scale III-Gait (Medication-Off/Stimulation-On vs. Medication-On/Stimulation-On)

The NMA results of the effect of the medication “on and off” on the mean change of the gait of the patients in stimulation-on are shown in Figure 5 and the SUCRA is shown in Figure 6. The comparison results of the NMA show that the GPi-DBS, STN-DBS, and PPN-DBS are superior to the baseline [GPi-DBS: –0.89(–1.1, –0.63), STN-DBS: –0.40(–0.52, –0.29), and PPN-DBS: –0.83(–1.5, –0.20)]. According to the results of the SUCRA, the GPi-DBS (SUCRA = 68.36%) ranks first, followed by PPN-DBS (SUCRA = 61.50%), and the STN-DBS (SUCRA = 20.03%) ranks last.

### 3.6. Unified Parkinson's Disease Rating Scale III-Total (Medication-Off/Stimulation-Off vs. Medication-Off/Stimulation-On)

**Figure 7:**
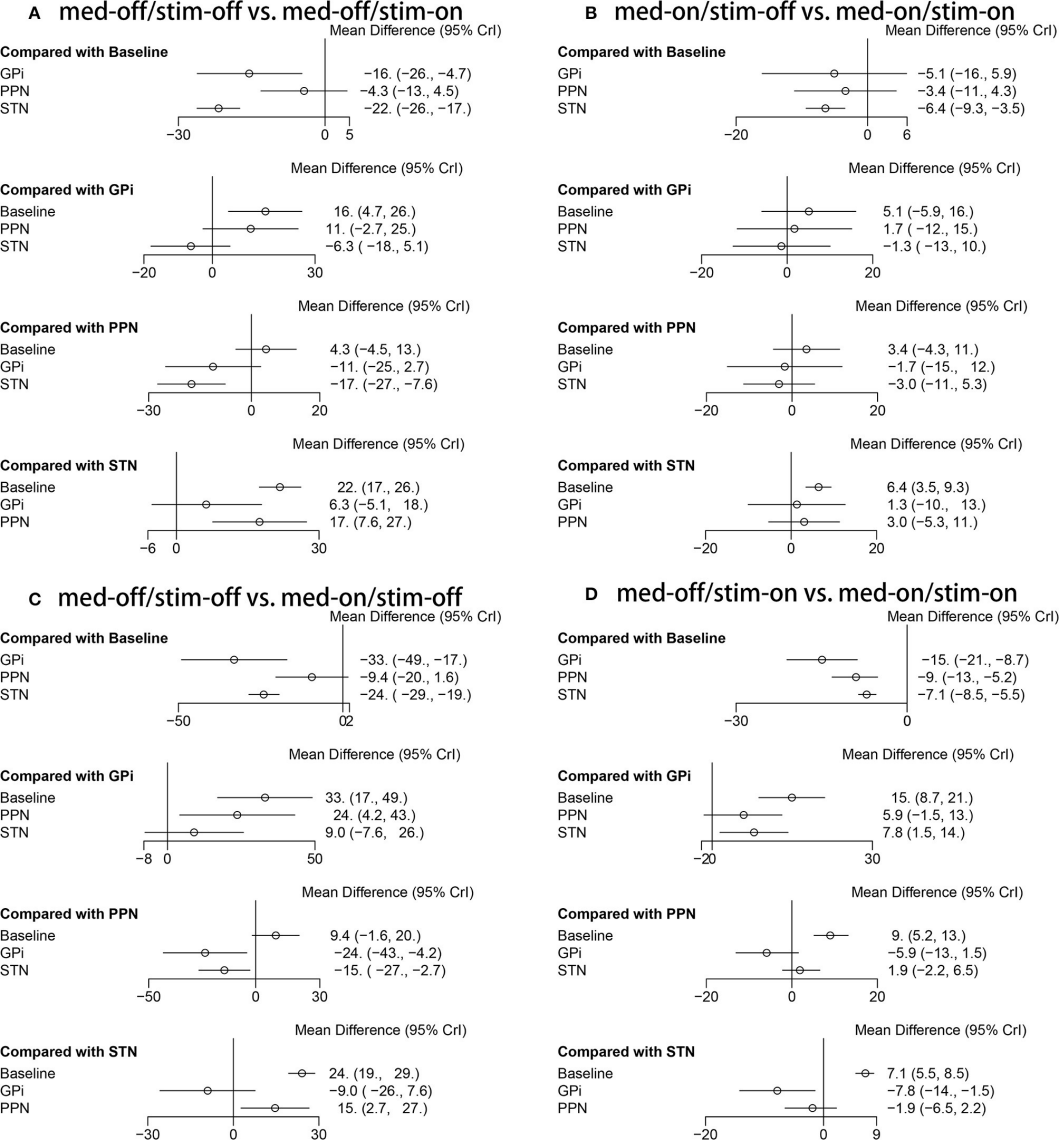
Forest plot of mean difference of UPDRS-III (Total); **(A)** med-off/stim-off vs. med-off/stim-on, **(B)** med-on/stim-on vs. med-on/stim-on, **(C)** med-off/stim-off vs. med-on/stim-off, **(D)** med-off/stim-on vs. med-on/stim-on.

**Figure 8:**
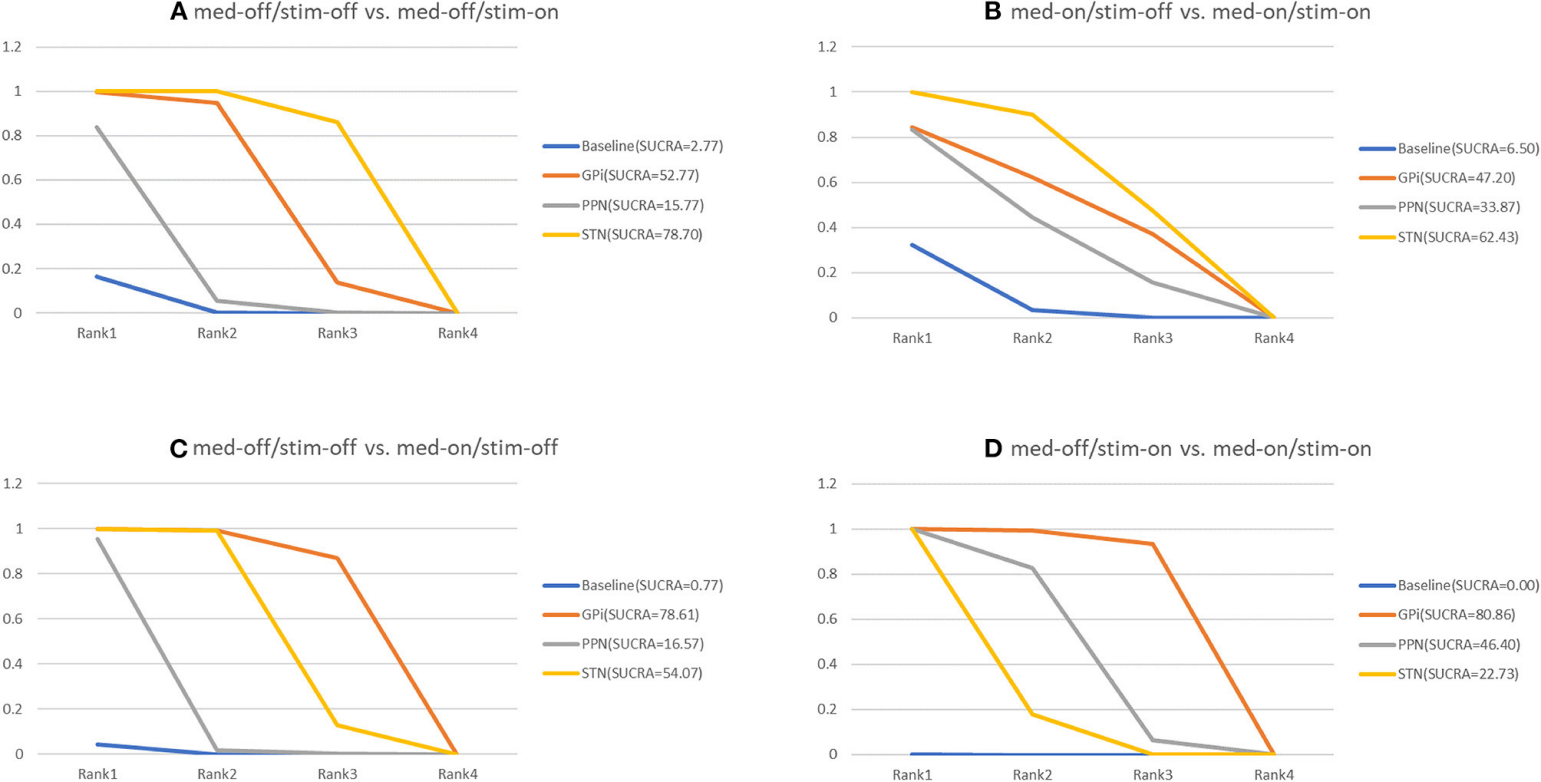
Cumulative rank of three surgical intervention of UPDRS-III (Total); **(A)** med-off/stim-off vs. med-off/stim-on, **(B)** med-on/stim-on vs. med-on/stim-on, **(C)** med-off/stim-off vs. med-on/stim-off, **(D)** med-off/stim-on vs. med-on/stim-on.

The secondary results of discontinuation are motor symptoms. The NMA results show the effect of stimulation “on and off” on the mean change of the gait of the patients in medication-off (Figure 7), and the SUCRA is shown in Figure 8. Compared with baseline, the GPi-DBS and STN-DBS proved to be significantly effective [GPi-DBS: –16.0(–26.0, –4.7) and STN-DBS: –22.0(–26.0, –17.0)]. The SUCRA scores reveal that the rank of the three surgical interventions is as follows: The STN-DBS (SUCRA = 78.70%) ranks first, followed by GPi-DBS (SUCRA = 52.77%), and PPN-DBS (SUCRA = 15.77%) ranks last.

### 3.7. Unified Parkinson's Disease Rating Scale III-Total (Medication-On/Stimulation-Off vs. Medication-On/Stimulation-On)

The NMA results of the effect of the stimulation “on and off” on the mean change of the gait of the patients in medication-on are summarized in Figure 7. In addition, the SUCRA is shown in Figure 8. The STN-DBS has effective improvement compared to the baseline [STN-DBS: –6.4(–9.3, –3.5)]. The SUCRA scores reveal that the rank of the three surgical interventions is as follows: the STN-DBS (SUCRA = 62.43%) ranks first, followed by GPi-DBS (SUCRA = 47.20%), and the PPN-DBS (SUCRA = 33.87%) ranks last.

### 3.8. Unified Parkinson's Disease Rating Scale III-Total (Medication-Off/Stimulation-Off vs. Medication-On/Stimulation-Off)

The NMA results of the effect of the medication “on and off” on the mean change of the gait of the patients in stimulation-off are summarized in Figure 7. In addition, SUCRA is shown in Figure 8. Compared with the baseline, the GPi-DBS and STN-DBS proved to be significantly effective [GPi-DBS: –33.0(–49.0, –17.0) and STN-DBS: –24.0(–29.0, –19.0)]. According to the SUCRA scores, the GPi-DBS (SUCRA = 78.61%) ranks first, followed by STN-DBS (SUCRA = 54.07%), and the PPN-DBS (SUCRA = 16.57%) ranks last.

### 3.9. Unified Parkinson's Disease Rating Scale III-Total (Medication-Off/Stimulation-On vs. Medication-On/Stimulation-On)

The NMA results of the effect of the medication “on and off” on the mean change of the gait of the patients in stimulation-on are shown in Figure 7 and the SUCRA is shown in Figure 8. The GPi-DBS, STN-DBS, and PPN-DBS show effective improvement compare to the baseline [GPi-DBS: –15.0(–21.0, –8.7), STN-DBS: –7.1(-8.5, –5.5), and PPN-DBS: –9.0(–13.0, –5.2)]. According to the SUCRA scores, the GPi-DBS (SUCRA = 80.86%) ranks first, followed by PPN-DBS (SUCRA = 48.40%), and the STN-DBS (SUCRA = 22.73%) ranks last.

### 3.10. Consistency and Integration Analysis, Small-Scale Research Effects

In this study, we assessed the inconsistency between the included studies by constructing a consistency model and an inconsistency model. The results show that the difference in DIC between these two models was less than 1. Thus, the consistency model is reliable. In addition, by limiting the value of all potential proportional reduction factors of different parameters to 1, it is demonstrated that the algorithm has good convergence efficiency. Moreover, we did not find a small-scale research effect.

## 4. Discussion

This study includes data from 34 clinical trials (538 patients) and systematically reviews the treatment of PD gait with different DBS targets and NMA. This study found that STN-DBS is the best treatment option to improve PD gait, while GPi-DBS is the best to improve PD gait under medication. STN-DBS ranked first in improving PD gait and motor symptoms and can greatly increase the stride length and increase the gait speed in the drug withdrawal state, while the rhythm remains mostly unchanged (Allert et al., 2001; Faist, 2001; Liu et al., 2005). At the same time, it also increases the swing motion amplitude of the arms and legs (Carpinella et al., 2007). STN- DBS also counteracts the asymmetry of foot position in pathological space, resulting in a more physiologically alternating gait cycle (Johnsen et al., 2009). Our findings are consistent with the findings of most previous studies. STN-DBS ranked first in improving the UPDRS III-29 gait score in the drug withdrawal state.

In principle, the effect of STN-DBS on gait parameters is similar to that of over-dose levodopa (L-dopa) (Cantiniaux et al., 2009; Hausdorff et al., 2009; Gulberti et al., 2015; Muthuraman et al., 2018), although L-DOPA sometimes has a slight impact on increasing step length and gait speed (Stolze et al., 2001; Lubik et al., 2006). Compared with any other treatment, the comprehensive effect of L-DOPA combined with STN-DBS is better than the effect of each alone (Hausdorff et al., 2009). In this study, the results of comparing the effects of L-dopa and STN-DBS are consistent with the general observation that STN-DBS can improve the symptoms of patients with dopamine-responsive PD (Pötter-Nerger and Volkmann, 2013). Our study found that GPi-DBS is more effective than STN-DBS in improving PD gait under medication. The effect of GPi-DBS on gait is different from that of STN-DBS. The GPi-DBS primarily affects gait speed, while STN-DBS primarily affects step length without changing the rhythm (Allert et al., 2001). Some studies have shown that GPi-DBS significantly improves the axial symptoms of untreated patients with PD in the first year after surgery, and this effect is not obvious under the state of drug treatment (Bakker et al., 2004). For gait and balance problems in patients with PD, the choice of GPi or STN as the target remains a matter of controversy.

A meta-analysis showed that when combined with drugs, the effect of STN-high-frequency stimulation (STN-HFS) on postural instability and gait disorder (PIGD) symptoms gradually worsened and reached the preoperative level within 2 years, while the effect of GPi-HFS combined with drugs remained stable over time (George et al., 2010). This may be due to the superiority of GPi-DBS over STN-DBS in the long-term efficacy of PIGD. However, the judgment on the relative benefit of GPi vs. STN surgery for PIGD must consider that GPi-HFS patients receive more levodopa than STN-HFS patients under combination therapy. Besides, there are few long-term studies evaluating GPi-HFS, and further randomized controlled trials and long-term follow-up trials are needed (George et al., 2010; Pötter-Nerger and Volkmann, 2013).

The PPN was introduced as a possible stimulation target for the treatment of gait disorders in patients with advanced PD (Broen et al., 2011). The PPN located in the midbrain and the upper bridge cover is classically identified by its main cholinergic neurons. Due to its extensive connections with other areas of the brain and spinal cord, the PPN is considered to be an important part of the “mesencephalic locomotor region” (MLR), which has been shown to be an upper spinal cord that can initiate and regulate movement (Ryczko and Dubuc, 2013). Plaha study (Plaha and Gill, 2005) shows that PPN-DBS has a possible therapeutic effect on gait disorders in patients with advanced PD. The previous published meta-analysis on the efficacy of PPN-DBS on PD gait indicates that PPN-DBS can greatly improve PD-related gait disorders (Lin et al., 2020). The results of this NMA show that PPN-DBS is effective in improving the gait score of UPDRS III-29 during the drug off period compared to baseline, but it ranks behind STN-DBS and GPi-DBS. In addition, PPN-DBS also ranks last in the improvement of the motor symptom score of patients with PD, which was not significant compared with the baseline. PPN-DBS is a promising therapy for axial motor deficits in PD, particularly gait freezing and falls (Thevathasan et al., 2017; Lin et al., 2020). The enrolled studies included patients with PD, which mainly included patients with stiffness, tremor, and postural gait disorder. Hence, the results of the study show the effectiveness in PPN-DBS lower than STN-DBS and GPi-DBS. The original data of the included article did not provide specific types of patients, and we could not further subgroup analysis to evaluate axial motor deficits in PD.

This study has certain limitations. First, in this study, age and sex were not taken into account, and the patient population that underwent different DBS targets were imbalanced. Second, the UPDRS III-gait is not very sensitive in detecting the gait improvement in patients with PD. Third, the evaluation of long-term efficacy requires further research studies. Fourth, the parameters of DBS were not considered, which caused a certain deviation. Finally, our conclusion involves indirect comparison and since NMA combines direct and indirect comparisons that include observational evidence, the inherent differences between trials was not considered and bias were added when assessing effects.

## 5. Conclusion

Although both STN-DBS and GPi-DBS can affect some aspects of PD gait disorder, the treatment of gait disorder in PD is still a challenge. Our study compared and ranked three stimulus targets for the treatment of PD, and the results can help clinicians choose reasonable treatment strategies for patients with PD.

## Data Availability Statement

The original contributions presented in the study are included in the article/Supplementary Material, further inquiries can be directed to the corresponding author.

## Author Contributions

TC contributed to the data processing. FL contributed to the data Extraction. GC contributed to the overall design. All authors contributed to the article and approved the submitted version.

## Conflict of Interest

The authors declare that the research was conducted in the absence of any commercial or financial relationships that could be construed as a potential conflict of interest.

## Publisher's Note

All claims expressed in this article are solely those of the authors and do not necessarily represent those of their affiliated organizations, or those of the publisher, the editors and the reviewers. Any product that may be evaluated in this article, or claim that may be made by its manufacturer, is not guaranteed or endorsed by the publisher.
