# Supplementary material for: Comparison of the Efficacy of Deep Brain Stimulation in Different Targets in Improving Gait in Parkinson's Disease: A Systematic Review and Bayesian Network Meta-Analysis
Source: Front Hum Neurosci. 2021 Oct 22;15:749722. doi: 10.3389/fnhum.2021.749722 (PMC8568957; doi:10.3389/fnhum.2021.749722)
Supplement: Supplementary file 1 [file Data_Sheet_1.DOCX]

Supplementary **Table S1.** Full electronic search strategy

| **Pubmed** |
| --- |
| ((((((((predict*[tiab] OR predictive value of tests[mh] OR score[tiab] OR scores[tiab] OR scoring system[tiab] OR scoring systems[tiab] OR observ*[tiab] OR observer variation[mh])))) AND (((((((((((((Idiopathic Parkinson's Disease[Title/Abstract]) OR Lewy Body Parkinson Disease[Title/Abstract]) OR Lewy Body Parkinson's Disease[Title/Abstract]) OR Primary Parkinsonism[Title/Abstract]) OR Parkinsonism, Primary[Title/Abstract]) OR Parkinson Disease, Idiopathic[Title/Abstract]) OR Parkinson's Disease[Title/Abstract]) OR Parkinson's Disease, Idiopathic[Title/Abstract]) OR Parkinson's Disease, Lewy Body[Title/Abstract]) OR Idiopathic Parkinson Disease[Title/Abstract]) OR Paralysis Agitans[Title/Abstract])) OR "Parkinson Disease"[Mesh])) AND ((((((((Brain Stimulations, Deep[Title/Abstract]) OR Deep Brain Stimulations[Title/Abstract]) OR Stimulation, Deep Brain[Title/Abstract]) OR Stimulations, Deep Brain[Title/Abstract]) OR Brain Stimulation, Deep[Title/Abstract]) OR Electrical Stimulation of the Brain[Title/Abstract])) OR "Deep Brain Stimulation"[Mesh])))) AND tremor[Title/Abstract] |

**Supplementary** **Table S2.** Evaluation of inconsistency.

|  | **Consistence Model fit (residual deviance)** | | | **Inconsistence Model fit (residual deviance):** | | |
| --- | --- | --- | --- | --- | --- | --- |
|  | **Dbar** | **pD** | **DIC** | **Dbar** | **pD** | **DIC** |
| **A. UPDRS III-Gait (med-off/stim-off vs. med-off/stim-on)** | 73.65434 | 62.15639 | 135.81073 | 73.61927 | 62.13354 | 135.75281 |
|  | 78 data points, ratio 0.9443, I^2 = 0% | | | 78 data points, ratio 0.9438, I^2 = 0% | | |
| **B. UPDRS III-Gait (med-on/stim-off vs. med-on/stim-on)** | 41.53011 | 35.39319 | 76.9233 | 41.58343 | 35.34516 | 76.92859 |
|  | 44 data points, ratio 0.9439, I^2 = 0% | | | 44 data points, ratio 0.9451, I^2 = 0% | | |
| **C. UPDRS III-Gait (med-off/stim-off vs. med-on/stim-off)** | 34.24379 | 30.48685 | 64.73064 | 34.26094 | 30.47397 | 64.73491 |
|  | 36 data points, ratio 0.9512, I^2 = 0% | | | 36 data points, ratio 0.9517, I^2 = 0% | | |
| **D. UPDRS III-Gait (med-off/stim-on vs. med-on/stim-on)** | 35.88467 | 27.10868 | 62.99335 | 35.83515 | 27.05763 | 62.89278 |
|  | 44 data points, ratio 0.8156, I^2 = 0% | | | 44 data points, ratio 0.8144, I^2 = 0% | | |
| **E. UPDRS III-Total (med-off/stim-off vs. med-off/stim-on)** | 39.82229 | 37.93954 | 77.76183 | 39.73102 | 37.89698 | 77.62799 |
|  | 42 data points, ratio 0.9481, I^2 = 0% | | | 42 data points, ratio 0.946, I^2 = 0% | | |
| **F. UPDRS III-Total (med-on/stim-off vs. med-on/stim-on)** | 32.21652 | 29.1078 | 61.32432 | 32.12471 | 29.1004 | 61.22511 |
|  | 34 data points, ratio 0.9475, I^2 = 0% | | | 34 data points, ratio 0.9448, I^2 = 0% | | |
| **G. UPDRS III-Total (med-off/stim-off vs. med-on/stim-off)** | 32.25602 | 28.7195 | 60.97552 | 32.23655 | 28.69491 | 60.93146 |
|  | 32 data points, ratio 1.008, I^2 = 4% | | | 32 data points, ratio 1.007, I^2 = 4% | | |
| **H. UPDRS III-Total (med-off/stim-on vs. med-on/stim-on)** | 48.55145 | 32.06322 | 80.61467 | 48.54379 | 32.00549 | 80.54928 |
|  | 48 data points, ratio 1.011, I^2 = 3% | | | 48 data points, ratio 1.011, I^2 = 3% | | |
